# Glucose-6-phosphate dehydrogenase deficiency and reduced haemoglobin levels in African children with severe malaria

**DOI:** 10.1186/s12936-016-1396-1

**Published:** 2016-07-07

**Authors:** Christian N. Nguetse, Christian G. Meyer, Ayola Akim Adegnika, Tsiri Agbenyega, Bernhards R. Ogutu, Peter G. Kremsner, Thirumalaisamy P. Velavan

**Affiliations:** Institute of Tropical Medicine, University of Tübingen, Wilhelmstrasse 27, 72074 Tübingen, Germany; Vietnamese-German Center for Medical Research, Hanoi, Vietnam; Centre de Recherches Médicales de Lambaréné, Lambaréné, Gabon; Department of Physiology, School of Medical Sciences, University of Science and Technology, Kumasi, Ghana; Departments of Child Health and Medicine, Komfo Anokye Teaching Hospital, Kumasi, Ghana; Centre for Clinical Research, Kenya Medical Research Institute, Kisumu, Kenya; Fondation Congolaise pour la Recherche Médicale, Brazzaville, Republic of Congo

**Keywords:** Glucose-6-phosphate dehydrogenase deficiency, African children, Severe malaria

## Abstract

**Background:**

Extensive studies investigating the role of host genetic factors during malaria associate glucose-6-phosphate dehydrogenase deficiency with relative protection. G6PD deficiency had been reported to associate with anti-malarial drug induced with haemolytic anaemia.

**Methods:**

A total of 301 Gabonese, Ghanaian, and Kenyan children aged 6–120 months with severe malaria recruited in a multicentre trial on artesunate were included in this sub-study. *G6PD* normal (type B), heterozygous (type A^+^) and deficient (type A^−^) genotypes were determined by direct sequencing of the common African mutations G202A and A376G. Furthermore, multivariate analyses were executed to associate possible contributions of G6PD deficiency with baseline haemoglobin levels, parasitaemia and with severe malarial anaemia.

**Results:**

Two hundred and seventy-eight children (132 females and 146 males) were successfully genotyped for *G6PD* variants. The overall prevalence of G6PD deficiency was 13 % [36/278; 3 % (4/132) female homozygous and 22 % (32/146) male hemizygous], 14 % (40/278) children were female heterozygous while 73 % (202/278) were G6PD normal [67 % (88/132) females and 78 % (114/146) males] individuals. Multivariate regression revealed a significant association of moderately and severely deficient *G6PD* genotypes with haemoglobin levels according to the baseline data (*p* < 0.0001; G6PD heterozygous: *p* < 0.0001; G6PD deficient: *p* = 0.009), but not with severe malarial anaemia (*p* = 0.66). No association of *G6PD* genotypes with baseline parasitaemia.

**Conclusions:**

In this study, moderately (type A^+^) and severely (type A^−^) G6PD deficiency showed significant association with lower haemoglobin concentrations at baseline in African children with severe malaria without leading to severe malarial anaemia. In addition, there was no association of *G6PD* variant types with parasite densities on admission.

## Background

Malaria remains a major health problem, with approximately 3.2 billion people at risk. In 2015, WHO reported approximately 214 million cases and about 438,000 deaths occurring in the world with the highest morbidity and mortality rates observed in Africa, especially among children under 5 years of age [1]. The control of the disease particularly in low transmission settings is the key target for malaria elimination [2] but such success is still a great challenge [3]. One approach to reduce the disease incidence is to block the transmission. Primaquine, a 8-aminoquinoline effective for both transmission blocking of *Plasmodium falciparum* and anti-relapse treatment against *Plasmodium vivax* has been recommended for many years by the World Health Organization (WHO) [4, 5]. However, primaquine has a major drawback which limits its widely use. The drug is known to cause acute haemolytic anaemia in individuals with glucose-6-phosphate-dehydrogenase (G6PD) deficiency [6, 7].

G6PD is a key enzyme catalysing the first reaction in the pentose phosphate pathway and provides a reduced form of nicotinamide adenine dinucleotide phosphate (NADPH) to help cells to counterbalance oxidative stress [8]. G6PD deficiency is a common, X-linked hereditary enzyme deficiency affecting approximately 400 million people worldwide [9], mainly in malaria-endemic regions [10]. Among the 186 mutations identified until 2012 in the *G6PD* gene [11], the variants 376A (G6PD type B), 376G (G6PD deficiency type A^+^ and 202A (severe G6PD deficiency type A^−^) are the most common ones. The deficiency types A^+^ (moderately deficient) and A^−^ (severely deficient) constitute up to 90 % of reported *G6PD* variants [8, 12, 13]. Other mutations such as A542T, G680T or T968C have also been identified in parts of Africa and been suggested to contribute to G6PD deficiency. However, information available on these mutations is very scarce. For example, the T968C mutation has been reported to be common only in The Gambia [14] and Senegal [15]. Although, G6PD diagnostic enzyme tests are available, they are currently not widely used in most clinical studies, particularly because primaquine, is not regularly used in Africa. Any attempts to control malaria need to take into account also tertian malaria, which, although occurring rarely only in many parts of Africa, contributes to the world-wide malaria burden.

Previous case–control studies have reported an association of G6PD deficiency, in particular of the 202A variant, with an increased risk of severe malarial anaemia and a protection or reduced risk against cerebral malaria [16, 17]. Therefore, it is interesting to assess the influence of this allele on the clinical presentation of severe malaria among the African children recruited into this study.

This study was a sub-study of a multicentre trial of artesunate conducted by the “Severe Malaria in African Children” (SMAC) consortium which assessed the non-inferiority of a simplified 3-dose regimen of intramuscular and intravenous artesunate [18]. The aim was to determine using direct sequencing the frequency distribution of G6PD deficiency in malaria children from Africa. Some exploratory analyses have also been performed to investigate the effect of *G6PD* genotypes on asexual parasitaemia, haemoglobin concentrations and severe malarial anaemia during admission.

## Methods

### Study design and participants

This study was a sub-study of the SMAC follow-up study. The SMAC study was an open-label, randomized, multicentre (Gabon, Malawi, Ghana, Kenya, and The Gambia), parallel-group, three-arm study to compare the anti-malarial activity and safety of three artesunate (ARS) dosing regimens in children with severe *P. falciparum* malaria. Patients were randomly assigned to one of three dosing regimens consisting of a total of 12 mg/kg parenteral ARS: (i) 2.4 mg/kg intramuscular on admission and at 12, 24, 48 and 72 h, (ii) 4 mg/kg intramuscular on admission and at 24 and 48 h, and (iii) 4 mg/kg intravenous on admission and at 24 and 48 h post admission. Parasitaemia was assessed by thick blood smears at 6 h intervals and prior to the each dose of treatment for at least 48 h following the first dose of study drug. Malaria occurs holoendemically and transmission rates in all study countries are high and perennial.

The present study involved participants of the SMAC study from Gabon, Ghana and Kenya. Three hundred and one children aged 6–120 months with a diagnosis of *P. falciparum* infection (parasitaemia ≥5000 parasites/µL on initial blood smear) made using an alternative to conventional thick film examination (Lambaréné method) [19] were randomly selected from the SMAC follow-up study [18]. Blood samples (400 μL) from all participants were collected in heparinized tubes. Specimens were stored at −80 °C for subsequent molecular analyses.

### Ethics statement

The study was conducted in accordance with Good Clinical Practices, and approved by authorities for each study site (the Regional Ethics Committee in Lambaréné (CERIL) for Gabon, Committee on Human Research, Publication and Ethics, Kwame Nkrumah University of Science and Technology (KNUST), Kumasi, for Ghana and the National Ethics Research Committee, Kenya Medical Research Institute (KEMRI) for Kenya). Children were enrolled into this study if a parent or guardian was willing to provide written informed consent in accordance with local practice.

### G6PD genotyping

Genomic DNA was isolated using QIAamp DNA mini blood kit (Qiagen, Hilden, Germany). A 968 bp fragment of the *G6PD* gene containing the polymorphisms 202G > A and 376A > G was amplified by PCR using primers 5′-GCCCCTGTGACCTCCCGCCA-3′ (forward) and 5′-GCAACGGCAAGCCTTACATCTGG-3′ (reverse). The main focus was directed only to these two variants although some other deficient genetic mutations such as A542T (Senegal 1 %, The Gambia 2.2 %), G680T (The Gambia 0 %, Senegal 0 %) and T968C (The Gambia 7.8 %, Senegal 10 %) have been reported at a substantially lower prevalences only [14, 15], and might have been present in this study population. However, they seem not to be responsible for the prevalence of G6PD deficiency in all parts of Africa [20]. Briefly, 10 ng of genomic DNA were added to a 20 µL reaction mixture containing 1 × PCR buffer (20 mM Tris–HCl pH 8.4, 50 mM KCl, 1.5 mM of MgCl_2_), 0.125 mM of dNTPs, 0.25 mM of each primer and 1 U Taq DNA polymerase (Qiagen, Hilden, Germany). The PCR was run on a PTC-200 Thermal cycler (MJ Research, Waltham, USA). Thermal conditions after initial denaturation (94 °C, 5 min) were 35 cycles of 94 °C for 45 s, 65 °C for 1 min, and 72 °C for 1 min. PCR reactions were completed with a final extension step of 72 °C for 5 min. PCR products were visualized through electrophoresis on a 1.2 % agarose gel stained with SYBR green I in 1x Tris-electrophoresis buffer (90 mM Tris–acetate, pH 8.0, 90 mM boric acid, 2.5 mM EDTA).

Subsequently, PCR products were purified (Exo-SAP-IT, USB, Affymetrix, USA) and directly used as templates for DNA sequencing using the BigDye terminator v. 1.1 cycle sequencing kit (Applied Biosystems, Foster City, USA) on an ABI 3130XL DNA sequencer. *G6PD* polymorphisms were identified by assembling the sequences with the reference sequence of *G6PD* (NG_009015.2) gene using the Codoncode Aligner 4.0 software (http://www.codoncode.com) and visually reconfirmed from their electropherograms.

### Statistical analysis

Data were analysed by using GraphPad Prism v. 5.0 for windows (GraphPad software, San Diego, CA). The effect of *G6PD* genotypes was determined on initial parasitaemia and haemoglobin values using a multivariate regression model. The children were classified in groups of normal, intermediate (female heterozygous) and deficient (hemizygous males and homozygous females) individuals. To evaluate the effect of *G6PD* genotypes on haemoglobin concentrations, the model included adjustment for age, gender, centre, weight, temperature and parasitaemia. To investigate the *G6PD* effect on parasitaemia, parasite densities were log-transformed and the model included adjustment for age, gender, centre, weight, haemoglobin levels and temperature. For the construction of the multivariate regression model, a subjective model-building approach that excludes possible confounders such as gender, age and origin of study participants was applied. Kruskal–Wallis of One-way ANOVA with Dunn’s Multiple Comparison and Mann–Whitney tests were used to determine the differences among categories. The level of significance was set to a *P* value of 0.05.

## Results

### Patients

**Fig. 1:**
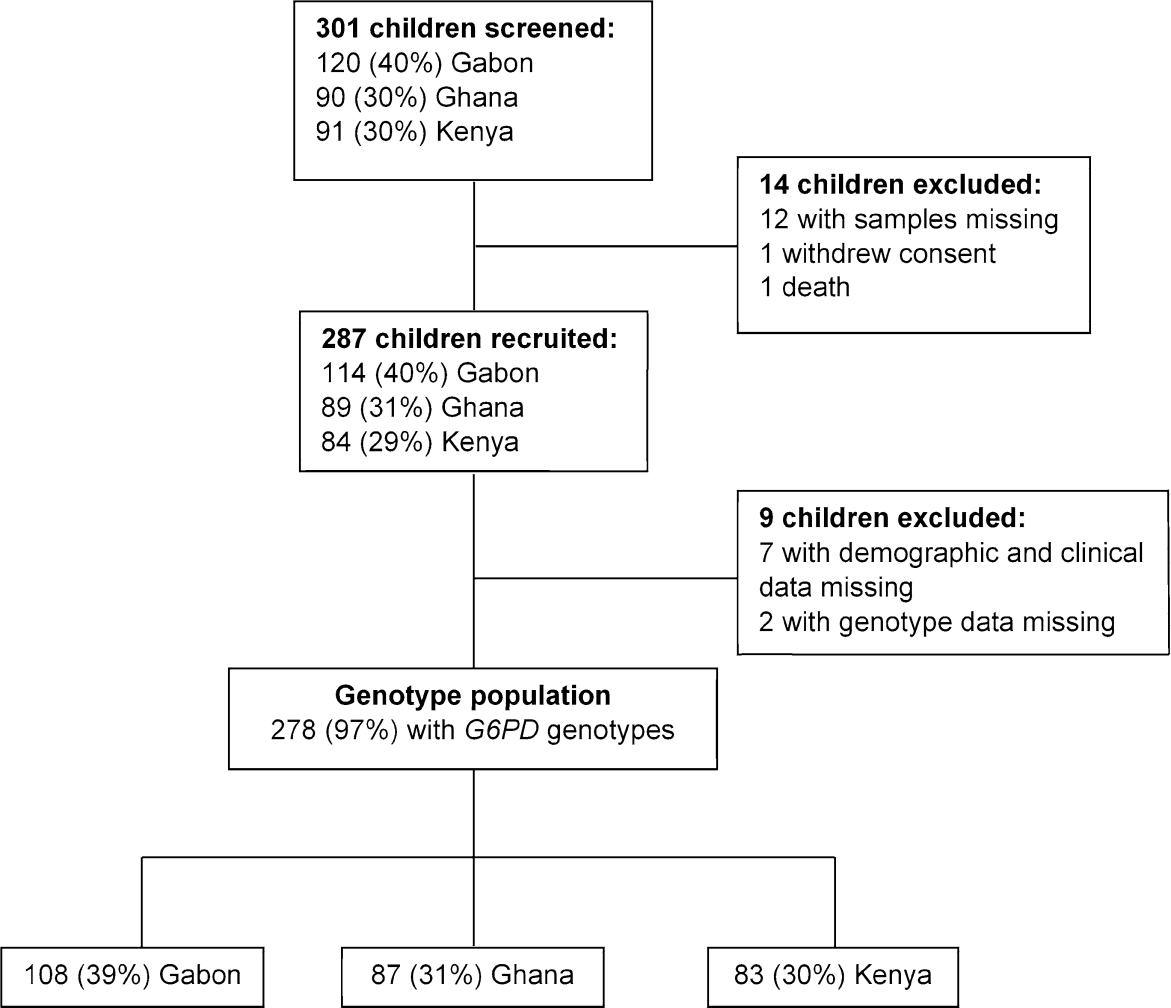
Patient population profile

According to the SMAC definition of severe malaria which perfectly reflects the policies of most African hospitals [21, 22], the frequency of severe malaria syndromes at presentation were substantially different across the three study sites (Table 1). The majority of children fulfilled one or more criteria of the WHO definition of severe malaria [23, 24], which include severe anaemia (haematocrit of <15 % or Hb <5 g/dL with a parasitaemia of >10,000/μL), hyperlactataemia (≥5 mmol/L), hyperparasitaemia (>250,000 parasites/μL), hypoglycaemia (whole blood or plasma glucose ≤2.2 mmol/L), and haemoglobinuria (urine that is dark red or black, with a dipstick that is positive for Hb/myoglobin). A description of children screened, recruited and genotyped for their G6PD status is shown in Fig. 1. From the 287 malaria children enrolled, the *G6PD* genotypes were available for only 278 children. One hundred and forty-six children (53 %) were males. The median age was 2 (IQR: 1–4) years ranging from 6 months to 10 years with a mean haemoglobin value of 8.5 (± 2.4) g/dL.

**Table 1:** Distribution of severe malaria syndromes by study centre

	All (%)	Lambaréné, Gabon (%)	Kumasi, Ghana (%)	Kisumu, Kenya (%)
Severe malaria syndromes at admission^a^				
Respiratory distress	10/278 (4)	1/108 (1)	9/87 (10)	0/83 (0)
Prostration	55/278 (20)	4/108 (4)	43/87 (49)	8/83 (10)
Cerebral malaria	13/278 (5)	0/108 (0)	12/87 (14)	1/83 (1)
General seizure	20/278 (7)	4/108 (4)	13/87 (15)	3/83 (4)
Severe anaemia	26/278 (9)	5/108 (5)	20/87 (23)	1/83 (1)
Jaundice	21/278 (8)	0/108 (0)	16/87 (18)	5/83 (6)

Children can appear in more than one category

^a^Missing data for some syndromes

### Prevalence of *G6PD* genotypes and associations with baseline variables

Overall, 202 (73 %) children were classified as G6PD normal [type B; 114 (78 %) males, 88 (67 %) females], while 40 (14 %) were female heterozygous (type A^+^) and 36 (13 %) were G6PD deficient [type A^−^; 32 (22 %) males hemizygous and 4 (3 %) female homozygous]. In Table 2, the genotype frequencies of *G6PD* for males and females by study site are shown. Between the study centres, there was a significant difference (*p* < 0.0001) in the prevalence of A^−^ G6PD deficiency among males. Among females, the frequency of A^−^ G6PD deficiency was lower compared to males. The prevalence was 4 % in Lambaréné (Gabon), 5 % in Kumasi (Ghana) and 0 % in Kisumu (Kenya).

**Table 2:** Frequency of *G6PD* genotypes in malaria children from the three study centres

Country	Centre	Male, N	B	A+	A−	Female, N	BB	BA+	A+A+	BA−	A+A−	A−A−
Gabon	Lambaréné	52	28 (54)	9 (17)	15 (29)	56	20 (35)	14 (25)	2 (4)	1 (2)	17 (30)	2 (4)
Ghana	Kumasi	46	25 (54)	8 (18)	13 (28)	41	10 (24)	11 (27)	2 (5)	1 (2)	15 (37)	2 (5)
Kenya	Kisumu	48	31 (65)	13 (27)	4 (8)	35	22 (63)	7 (20)	0 (0)	0 (0)	6 (17)	0 (0)
Total		146	84 (58)	30 (20)	32 (22)	132	52 (39)	32 (24)	4 (3)	2 (2)	38 (29)	4 (3)

Data are shown as N (%). G6PD genotype: male normal = A+ or B; male hemizygous = A−; female normal = BB or BA+ or A+A+; female heterozygous = BA− or A+A−; female homozygous = A−A−

To investigate the association of *G6PD* genotypes with baseline variables, the children were grouped based on gender and *G6PD* genotype. Baseline demographic and clinical data are given in Table 3. Using a multivariate regression analysis adjusted for age, gender, centre, weight, temperature and parasitaemia, there was a significant association of the *G6PD* genotypes with the adjusted mean baseline of haemoglobin concentrations (*p* < 0.0001). Furthermore, a comparison between children G6PD normal and heterozygous (*p* < 0.0001), and between individuals G6PD normal and G6PD deficient (*p* = 0.009) showed a significant difference in adjusted mean baseline haemoglobin. The *G6PD* mutant genotypes were associated with a 2.7 g/dL and 1 g/dL decrease in haemoglobin levels in heterozygous and deficient children, respectively. The multivariate regression analysis adjusted for age, gender, centre, weight, haemoglobin levels and temperature did not result in any association (*p* = 0.29) of the *G6PD* genotypes on adjusted mean baseline parasite densities. In addition, there was no difference in adjusted log mean parasite densities between G6PD normal and heterozygous individuals (*p* = 0.18), and between G6PD normal and deficient children (*p* = 0.07).

**Table 3:** Baseline demographic and clinical data following *G6PD* genotypes

Characteristic	Male normal (n = 114)	Male hemizygous (n = 32)	Female normal (n = 88)	Female heterozygous (n = 40)	Female homozygous (n = 4)	All children (n = 278)
Age, years [range]	3.3 (2.8) [0–10]	2.6 (2.2) [0–8]	2.2 (1.9) [0–8]	2.3 (2.6) [0–10]	2.5 (3) [0–6]	2.7 (2.5) [0–10]
0–3, n (%)	65 (57)	23 (72)	69 (78)	30 (75)	2 (50)	189 (68)
4–7, n (%)	39 (34)	8 (25)	18 (21)	7 (18)	2 (50)	74 (27)
≥8, n (%)	10 (9)	1 (3)	1 (1)	3 (7)	0 (0)	15 (5)
Weight, kg	15.6 (5.2)	12.6 (4.7)	13.2 (4)	13.2 (5.9)	16.3 (2.7)	14.1 (5)
Parasitemia per μL, geometric mean [range]	150,464 [6240–1,270,080]	95,369 [8153–709,400]	138,296 [5377–1,677,780]	105,520 [6380–745,100]	172,340 [32,176–397,440]	132,349 [5377–1,677,780]
Temperature, °C	37.9 (1.1)	38.4 (1)	38.2 (1.1)	38 (1.2)	37.7 (1.3)	38.1 (1.1)
Haemoglobin, g/dL (years)	8.9 (2.4)	7.8 (2.3)	8.9 (2.3)	7.1 (2.3)	8.2 (1.4)	8.5 (2.4)
0–3	8.2 (2.4)	7.6 (2.1)	8.6 (2.3)	6.7 (2.2)	8.1 (2.1)	8.1 (2.4)
4–7	9.9 (2)	8.2 (2.8)	9.7 (1.8)	6.9 (2)	8.3 (1.3)	9.3 (2.2)
≥8	9.8 (1.8)	NA	NA	10.6 (0.6)	NA	10.2 (1.7)
Haematocrit, %	27.4 (7.3)	23.8 (7.1)	27.3 (7)	21.7 (7.6)	25.5 (4)	26.1 (7.5)
Red blood cell count, 10^12/L	3.8 (1)	3.3 (1.2)	3.6 (1)	3 (1)	3.1 (0.4)	3.6 (1.1)
Platelet count, 10^9/L	151.6 (230.4)	98 (82)	117.3 (118.8)	111 (77.7)	63.3 (30)	127.5 (167.9)
White blood cell count, 10^9/L	9.4 (4.2)	11.6 (7.9)	9.8 (4.3)	11.6 (4.4)	7.7 (3.4)	10.1 (4.9)

Values are mean (SD) [range] unless otherwise indicated. G6PD genotype: male normal = A or B; male hemizygous = A−; female normal = B/B or B/A or A/A; female homozygous = A−/A−; female heterozygous = B/A− or A/A−

*NA* not applicable; *SD* standard deviation

### Severe malarial anaemia and cerebral malaria

Following the WHO guidelines [23] which defines severe malarial anaemia (SMA) as Hb <5 g/dL, 26 children (9 %) were affected by SMA in this study. The mean haemoglobin concentrations was 4 g/dL (range 1.9–4.9 g/dL). There was no difference (*p* = 0.66) of the haemoglobin levels between SMA children with different *G6PD* genotypes. However, SMA occurred more frequently among G6PD normal 15/26 (58 %) compared to G6PD heterozygous 8/26 (31 %) and G6PD deficient 3/26 (12 %) children.

Only 13 patients (5 %) had cerebral malaria. Nine of them were G6PD normal and four were female G6PD heterozygous.

## Discussion

Glucose-6-phosphate dehydrogenase deficiency has raised in frequencies in malaria-endemic settings as a consequence of the evolutionary pressure exerted by malaria on the human genome [16]. A plethora of previous studies have indicated and suggested a correlation between malaria endemicity and the occurrence of G6PD deficiency (reviewed in [25]).

The main objective of this study was to assess the distribution of *G6PD* genotypes among African children from three geographically countries presenting with severe malaria and participating in the SMAC clinical trial on different artesunate treatment regimens. While meanwhile many *G6PD* variants have been described [8, 11], the main focus was on the most relevant three variants (376A (G6PD type B, no deficiency), 376G (moderate G6PD deficiency type A^+^) and 202A severe G6PD deficiency type A^−^) in Africa [26].

The prevalence of severe G6PD deficiency as determined genetically was higher compared to previous findings from Gabon with 17 % in the present study *versus* 14 % reported earlier among males and 7 vs 2 % among females [27]. In Ghana 28 % were observed in this study, compared to 9 % among males and 5 vs 3 % among females indicated previously [28]. However, in Kenya the prevalence of severe G6PD deficiency among females was lower compared to previous reports with 0 vs 5 % and equal among males 8 vs 8 % [29]. Regardless of the site, G6PD deficiency was considerably higher among males (22 %) compared to females (3 %), with an overall prevalence of 13 % across sites. This finding confirms that males are affected by this blood disorder rather than females and that G6PD deficient females are rather uncommon [26].

A significant association of moderately and severely deficient *G6PD* genotypes and haemoglobin levels according to the baseline data was observed. In fact, compared to children with the normal *G6PD* genotype (haemoglobin median: 9.3 g/dL), *G6PD* heterozygous (haemoglobin median: 6.6 g/dL) and deficient (haemoglobin median: 8.3 g/dL) children had 2.7 and 1 g/dL lower haemoglobin concentrations, respectively. The results contradict previous findings, which did not observe any association between *G6PD* genotypes and haemoglobin levels [30–32]. While in their study, May et al. [32] found lower levels of haemoglobin in *G6DP* deficient individuals, the association was not significant. An explanation could be the different designs of that and the present study. Here, patients with severe malaria were included, which implies high parasitaemia and haemoglobin concentrations <5 g/dL, whereas in the above cited study, the authors excluded patients with haemoglobin concentrations ≤7 g/L [30] and recruited individuals only with uncomplicated malaria [31] or who were asymptomatically infected [32].

In the case of SMA, there was no difference of haemoglobin levels among children with different *G6PD* genotypes. This may suggest that G6PD deficiency is not associated with this complication. However, this is in fact surprising especially as a significant difference of baseline haemoglobin values between G6PD normal and deficient individuals was observed. Moreover, in a large case–control study in Kenyan individuals, a significant increased risk to severe malarial anaemia associated with lower haemoglobin levels in G6PD deficient children with severe malaria at the time of hospital admission was found [33]. The likely explanation could be due to the sample size which was rather small.

Although not significant, parasite densities were lower in G6PD deficient children than in G6PD heterozygous, compared to G6PD normal individuals. However, this trend is in agreement with previous works [34, 35]. Conflicting results have been reported regarding parasitaemia and the various *G6PD* genotypes. Other studies have indicated a significantly lower parasitaemia associated with female G6PD heterozygous individuals, suggesting a protective advantage by this genotype [36–39].

In comparison to individuals with uncomplicated malaria, this study population was hyperparasitemic according to the criteria of severe malaria provided by WHO with lower parasitaemia in G6PD deficient individuals. However, either in uncomplicated or in severe/complicated malaria, G6PD deficiency was always associated with lower parasitaemia [30, 33]. Although the underlying genetic mechanisms are not completely clear, mechanisms suggested are impaired growth of *P. falciparum* parasites in G6PD deficient red blood cells [40] and slow rates of parasite replication [41, 42], more efficient clearance of infected red blood cells [43], and lower abundance of *P. falciparum* 6-phosphogluconolactonase mRNA in parasites from G6PD deficient individuals [44] both uncomplicated and severe malaria.

Several limitations apply to this study. Although some of the observations are in agreement with previous findings, the study group is rather small. The focus was only on the *G6PD* mutations 202G > A and 376A > G, although other variants such as A542T, G680T or T968C have also been reported to contribute to G6PD deficiency, albeit at far lower frequencies. The associations observed between *G6PD* genotypes and the baseline clinical parameters at admission could in fact also be the result of different circumstances. First, only patients with severe malaria were included, which could explain the findings, especially as the *G6PD* deficient allele 202A appears to confer protection against cerebral malaria and increases the risk of severe malarial anaemia [16, 17]. Second, depending on the mechanism of protection, G6PD deficiency might be associated with delayed presentation to the hospital and be a plausible explanation of the differences observed in haemoglobin concentrations at first admission. Third, alpha-thalassaemia, another haemoglobinopathy which has been associated with lower haemoglobin levels in alpha-thalassaemic Nigerian children and adults [45] cannot be ruled out as a confounding variable as it was not investigated in this study. The statistical analysis using multivariable regression model is in part subjective due to the adjusted variables added.

## Conclusions

G6PD deficiency is more common among children from Gabon and Ghana than in Kenya. A significant association of the *G6PD* genotypes studied to lower haemoglobin levels was observed, suggesting a possible contributions of G6PD deficiency to the reduced production of erythrocytes in affected individuals. This was, however, not related to severe malarial anaemia experienced by some children. There was no evidence of a significant association between lower parasitaemia observed in G6PD deficient individuals compared to normal ones.

## Abbreviations

G6PD: glucose-6-phosphate dehydrogenase
ARS: artesunate
SMAC: severe malaria in African children
WHO: World health organization
NADPH: nicotinamide adenine dinucleotide phosphate
SMA: severe malarial anaemia
